# Rooster Behavior and Laying Breeder Performance in Natural Mating Cages as a Function of Different Rearing Management

**DOI:** 10.3390/ani15131925

**Published:** 2025-06-30

**Authors:** Yuqi Chen, Yalan Zuo, Aosui Zhao, Yao Zhang, Shunshun Han, Can Cui, Huadong Yin

**Affiliations:** 1 Key Laboratory of Livestock and Poultry Multi-Omics, Ministry of Agriculture and Rural Affairs, College of Animal Science and Technology, Sichuan Agricultural University, Chengdu 611130, China; chenyuqi1@stu.sicau.edu.cn (Y.C.); zuoyalan@stu.sicau.edu.cn (Y.Z.); zhaoaosui@stu.sicau.edu.cn (A.Z.); zhangyao@sicau.edu.cn (Y.Z.); hanshunshun@sicau.edu.cn (S.H.); cuican123@stu.sicau.edu.cn (C.C.); 2 Farm Animal Genetic Resources Exploration and Innovation Key Laboratory of Sichuan Province, Sichuan Agricultural University, Chengdu 611130, China

**Keywords:** behavior, stocking density, sex ratio, natural mating colony cage, cohabitation age

## Abstract

Natural mating colony cages are crucial in poultry breeding, yet breed-specific management requires further investigation. We evaluated the effects of sex ratios, stocking densities, and cohabitation age on Lohmann Pink-shell breeders’ performance. A total of 6126 birds were randomly allocated to experimental groups with varying ratios (1:8–1:13), densities (582–748 cm^2^/bird), and cohabitation ages (120/140 days), each containing six replicates. We monitored male mating frequencies at 50 weeks in 1:8 and 1:10 ratio groups. All 120-day-old groups showed delayed production onset and superior male weight compliance (*p* < 0.01), with reduced egg breakage and increased healthy chick output (*p* < 0.01). Lower stocking densities (748/694 cm^2^/bird) showed lower breakage rate and uniformity than 582 cm^2^/bird (*p* < 0.05). The 1:10 sex ratio achieved optimal egg production and fertilization rate (*p* < 0.05). Male mating peaked between 16:00 and 18:00. Optimal parameters were 120-day age of cohabitation, 694 cm^2^/bird density, and 1:10 sex ratio, providing theoretical guidance for natural mating colony cage development in layer breeding.

## Simple Summary

Natural mating colony cages are the primary system for commercial layer breeder management, yet breed-specific optimization approaches remain insufficiently investigated. This study systematically evaluated the effects of sex ratios, stocking densities, and cohabitation age on reproductive performance in Lohmann Pink-shell breeders under cage-based natural mating conditions. Using a factorial design, we measured fertilization rate, hatchable egg rate, and reproductive stability across experimental groups. Results demonstrated that optimal parameters were 120-day cohabitation age, 694 cm^2^/bird stocking density, and 1:10 sex ratio, offering theoretical foundations for natural mating colony cage development in layers.

## 1. Introduction

Natural mating colony cage systems are increasingly adopted in commercial breeder operations due to their operational efficiency, low energy consumption, and environmentally sustainable practices. Growing concerns exist regarding hen welfare in conventional cage systems. Unlike traditional configurations, these colony cages co-house layer breeders with roosters, functioning as parent stock for commercial laying hens. Nevertheless, persistent challenges, including behavioral abnormalities and the necessity for optimized feeding management protocols, suggest that this technology remains in its nascent stage.

The implementation of vertically stacked natural mating colony cages is becoming a predominant housing paradigm for commercial layer breeders in China. This innovative system facilitates natural mating behaviors by maintaining mixed-sex flocks, prioritizing animal welfare while achieving production efficiency, energy conservation, and ecological sustainability [1]. Comparative studies on mating modalities demonstrate superior growth performance in naturally mated broilers, with reduced pathogen contamination and microbial loads [2]. Current breeding guidelines recommend sex ratios ranging from 1:8 to 1:12 across commercial layer varieties and production phases [3]. However, field applications often fail to achieve theoretical performance benchmarks, potentially influenced by daily husbandry practices in layer breeder management.

Existing research indicates that enlarged group sizes induce serum hormonal fluctuations associated with social stress, subsequently impacting production metrics [4]. Cohabitation age significantly influences avian growth trajectories and subsequent reproductive outcomes. During flock integration, social hierarchy formation precipitates increased aggressive interactions and locomotory activity, elevating metabolic demands. Although intraspecific aggression diminishes post-hierarchy establishment in hens, rooster conflict persists. Empirical evidence demonstrates that higher male-to-female ratios exacerbate hen stress levels, with elevated mating frequencies correlating with physiological and behavioral stress markers [5]. Notably, hen presence enhances rooster reproductive efficacy, particularly in semen quality parameters [6]. Nevertheless, systematic investigations into optimized management protocols for Lohmann Pink-shell breeders in colony cage systems remain limited, constraining full genetic potential realization. Critical rearing parameters, including stocking density, substantially affect pullet immunocompetence and ethological profiles [7].

Previous investigations predominantly focused on singular factors (sex ratio/density) in floor-reared systems [8,9]. This study comprehensively evaluates the combined effects of cohabitation age, sex ratio, and stocking density on production performance and rooster behavioral patterns in Lohmann Pink-shell layers under natural mating colony cage conditions.

## 2. Materials and Methods

### 2.1. Animal Ethics Statement

All experimental protocols in this research were approved by the Animal Welfare Committee of Sichuan Agricultural University under the approval number 20222102018.

### 2.2. Animals, Diets, and Experimental Design

A total of 6126 Lohmann Pink-Shell breeders (17-week-old, there is no statistically significant difference in body weight among birds of the same sex) were enrolled in this study. Experimental cages (supplied by BIG DUTCHMAN Co., Ltd., Vechta, Germany, 4800 × 1200 × 800 mm) were installed at Sichuan Guangyuan Shengruida Poultry Industry Co., Ltd., Guangyuan, China. All birds were housed on the same floor within the same facility. Birds received standardized nutritional regimens across the following growth phases:

5–15 weeks: Crude protein: 18.5–15%, ME: 2800 kcal/kg;

16–18 weeks: Crude protein: 17.5%, ME: 2750 kcal/kg;

19 weeks to endpoint: Crude protein: 18.5%, ME: 2750 kcal/kg.

The management protocol included four daily feed allocations with ad libitum water access. Environmental conditions were maintained at 22 ± 1 °C with 65% relative humidity, under controlled photoperiod (16L:8D cycle).

Prior to cohabitation, roosters and hens are maintained in individual rearing cages at group sizes of 8–10 birds per cage from 5 to 15 weeks of age (male cage: 600 × 600 × 500 mm; female cage: 600 × 600 × 450 mm, for 5 wk to 15 wk). The transition to natural mating colony cages typically occurs between 15 and 20 weeks.

Cohabitation age (the age at which roosters are added to the flock): A total of 1080 Lohmann Pink-shell breeders (120 roosters, 960 hens; 17-week-old) with uniform body weights were allocated from a single cohort. Two experimental groups were established: Group I (cohabitation initiated at 120 days) and Group II (140 days), each containing 6 replicates (90 birds/replicate; 10 roosters: 80 hens/cage). Rooster body weights were measured pre-cohabitation and 7 days post-introduction. Egg production metrics were recorded from day 10 post-cohabitation.

Stocking density: The density trial utilized 2076 birds (17-week-old) from a common source cohort. Four experimental configurations were tested:

Group III: 748 cm^2^/bird (77 birds/cage);

Group IV: 694 cm^2^/bird (82 birds/cage);

Group V: 654 cm^2^/bird (88 birds/cage);

Control group: 582 cm^2^/bird (99 birds/cage).

All groups maintained six biological replicates under standardized conditions.

Sex ratio: Five mating ratios (1:8, 1:9, 1:10, 1:11, 1:13) were evaluated using 2970 birds (17-week-old). Each ratio group comprised six replicated cages (99 birds/cage), with rooster: hen proportions adjusted per experimental design (e.g., 1:8 = 11 roosters:88 hens).

### 2.3. Breeding Rooster Behavior

At 50 weeks old, roosters were selected for the experimental groups with male-to-female ratios of 1:8 and 1:10 (according to the difference in fertilization rate between 25 and 45 wk). Prior to this experiment, each rooster was weighed and marked on its wings based on weight ranking. Two monitors (1080P, Xiaomi Co., Ltd., Beijing, China) were installed on the top of each cage to capture the movement of the flock. The collected videos were analyzed to record the primary activities of the roosters during each period.

### 2.4. Data Collection

Production metrics were systematically measured starting at 10 days post-cohabitation, encompassing mortality rate, weight control ratio (body weight/standard weight), flock uniformity (proportion of birds within 90–110% of mean body weight), average weekly gain (AWG), relative growth rate (RGR), and settable egg production (SEP, calculated as the percentage of eggs meeting corporate quality standards after excluding substandard specimens with inadequate weight (<47 g) or shell defects). From weeks 25–45 post-mixing, reproductive performance was assessed through weekly egg collection and hatching indices calculation, including fertilization rate, hatchability, hatchability of settable eggs (HS), and female chick viability rate (FCVR; healthy day-old pullets).

### 2.5. Statistical Analysis

The results were presented as mean ± standard error (SEM) and visualized using GraphPad Prism 8 software. Statistical analyses were conducted using SPSS 20 software. Student’s *t*-test was employed for parametric data, and non-parametric data was analyzed using non-parametric tests for comparisons between two groups. One-way analysis of variance (ANOVA) followed by Tukey’s test was utilized for comparisons among multiple groups. Significance levels were denoted as ** *p* < 0.01, * *p* < 0.05, lowercase letters (a, b, c) for *p* < 0.05, and capital letters (A, B, C) for *p* < 0.01.

## 3. Results

### 3.1. Effect of Cohabitation Age on Performance of Lohmann Pink Egg Breeder

The influence of cohabitation age on reproductive initiation in breeder hens is detailed in Table 1. The 120 and 140 groups reached 50% egg production at 156.65 and 155.05 days, respectively, with no statistically significant difference between them (*p* > 0.05).

Turning to the roosters, Table 1 also reveals notable differences in growth performance based on mixing age. Specifically, the 120-day-old cohabitation roosters exhibited a significantly higher relative growth rate (RGR), weekly weight gain (AWG), and body weight control rate compared to the 140-day group (*p* < 0.01). The 140-day group showed a higher body weight uniformity compared to the 120-day group(*p* < 0.01).

As quantified in Table 2, no statistically significant differences were observed in settable egg production (SEP) and laying rate between the two groups. Similarly, there was a statistically insignificant increase in egg breakage in the 140-day group compared to the 120-day group. Egg weights were not statistically different at other weeks of age; the 120-day cohort exhibited significantly heavier eggs at week 37 (*p* < 0.05). Mortality rates showed no treatment-associated differences between groups for either sex.

As detailed in Table 3, the two cohorts showed comparable outcomes for healthy female chick production (HFCP) and Fertility. However, at 33 weeks, the 120-day group demonstrated significantly higher overall hatchability compared to the 140-day group (*p* < 0.05), with hatchability of settable eggs (HSE) showing a marked improvement (*p* < 0.01). These parameters showed no statistical differences at other developmental stages.

### 3.2. Effect of Breeding Density on Performance of Lohmann Pink-Egg Breeder

Table 4 summarizes the effects of stocking density variations on Lohmann Pink layer breeders in colony cage systems. Laying rates in intervention groups were significantly reduced relative to controls (582 cm^2^/bird), with Group III (748 cm^2^/bird) showing the most pronounced decline (*p* < 0.05). Laying performance remained comparable between other intervention groups (IV and V).

Production performance revealed uniform egg breakage rates across all groups. Group IV demonstrated significantly higher settable egg production (SEP) than Group III (*p* < 0.05), though no differences emerged when compared with other cohorts. Egg weights in intervention groups were significantly reduced compared to controls (*p* < 0.05). Mortality rates remained statistically equivalent across all cohorts for both sexes.

Growth analysis showed that control hens achieved superior weight regulation (108.6%), significantly exceeding intervention groups (*p* < 0.05). Intervention groups showed comparable weight regulation indices among themselves. Group III hens displayed significantly improved flock weight uniformity versus controls (*p* < 0.05), though no inter-group variations reached significance. Rooster weight showed no treatment effects. Notably, Group III achieved peak uniformity in roosters (98.15%), significantly exceeding control levels (*p* < 0.05).

Hatching performance (fertility, hatchability, HS, and FCVR) did not differ significantly among density groups (*p* > 0.05).

### 3.3. Effect of Sex Ratio on Production Performance and Fertility Rate

As demonstrated in Table 5, the 1:8 sex ratio group showed significantly reduced laying rates compared to the 1:9 group (*p* < 0.05). The 1:10 group exhibited the lowest egg breakage incidence, with a statistically significant reduction relative to the 1:9 group (*p* < 0.05). Settable egg production (SEP), death and culling rate of hens (DCH), and death and culling rate of roosters (DCR) remained comparable across all groups. Notably, the 1:10 group achieved significantly enhanced fertilization rates compared to the 1:8 group (*p* < 0.05).

To assess temporal fertilization patterns under varying sex ratios, longitudinal measurements were conducted as detailed in Table 6. This analysis revealed stage-specific variations:

At 25 weeks: 1:10 > 1:8 (*p* < 0.05);

At 29 weeks: 1:8, 1:10, 1:13 > 1:9 (*p* < 0.05);

At 37 weeks: 1:11, 1:13 > 1:8 (*p* < 0.05);

At 41 weeks: 1:10 > 1:8 (*p* < 0.05);

At 45 weeks: 1:10, 1:11 > 1:13 (*p* < 0.05).

No significant intergroup differences emerged at 33 weeks. Cumulatively, the 1:10 ratio demonstrated sustained fertilization superiority over 1:8 (*p* < 0.05), while other ratios showed comparable performance.

**Table 5:** Effect of different sex ratios on production performance of laying breeders (25–45 wk).

Sex Ratio	1:8	1:9	1:10	1:11	1:13	*p*-Value
Laying rate (%)	90.72 ± 1.61 ^b^	93.64 ± 1.74 ^a^	92.68 ± 2.42 ^ab^	91.57 ± 2.49 ^ab^	92.10 ± 3.35 ^ab^	0.021
Breakage rate (%)	1.38 ± 0.12 ^ab^	1.44 ± 0.16 ^a^	1.08 ± 0.19 ^b^	1.13 ± 0.15 ^b^	1.39 ± 0.18 ^ab^	0.013
SEP ^1^ (%)	93.72 ± 1.11	94.34 ± 3.23	94.44 ± 3.18	95.44 ± 2.47	94.15 ± 3.92	0.651
DCH ^2^ (%)	0.11 ± 0.13	0.03 ± 0.09	0.11 ± 0.19	0.18 ± 0.22	0.11 ± 0.12	0.517
DCR ^3^ (%)	0	1.00 ± 1.10	0	0.83 ± 1.29	0	0.894
Fertility (%)	91.65 ± 1.62 ^b^	91.85 ± 0.82 ^ab^	93.40 ± 1.11 ^a^	92.43 ± 1.80 ^ab^	92.02 ± 1.13 ^ab^	0.027

^a,b^ Means in the same line with different superscripts are different (*p* < 0.05); ^1^ SEP: Percentage of settable eggs; ^2^ DCH: Death and culling rate of hens; ^3^ DCR: Death and culling rate of roosters.

**Table 6:** Trend of fertility percentages of different sex ratios (25–45 wk).

Sex Ratio	25 wk	29 wk	33 wk	37 wk	41 wk	45 wk
1:8	84.56 ± 1.74 ^b^	91.19 ± 1.49 ^a^	92.46 ± 0.40	90.67 ± 0.60 ^b^	91.07 ± 2.01 ^b^	93.06 ± 0.82 ^ab^
1:9	87.20 ± 2.55 ^ab^	88.76 ± 1.15 ^b^	92.65 ± 0.79	92.99 ± 1.22 ^ab^	92.99 ± 1.62 ^ab^	94.97 ± 1.04 ^ab^
1:10	88.47 ± 2.12 ^a^	91.94 ± 0.67 ^a^	92.83 ± 1.08	92.93 ± 1.07 ^ab^	93.86 ± 1.04 ^a^	95.18 ± 0.91 ^a^
1:11	85.28 ± 2.14 ^ab^	90.86 ± 1.26 ^ab^	93.37 ± 0.67	94.18 ± 1.21 ^a^	92.99 ± 1.20 ^ab^	95.09 ± 0.16 ^a^
1:13	87.07 ± 1.97 ^ab^	91.52 ± 1.46 ^a^	93.65 ± 1.28	94.05 ± 1.69 ^a^	92.59 ± 0.73 ^ab^	92.46 ± 0.93 ^b^
*p*-value	0.016	0.027	0.604	0.032	0.027	0.015

^a,b^ Means in the same column with different superscripts are different (*p* < 0.05).

### 3.4. Correlation Analysis of Mating Behavior of the Roosters in This Breeding Cage

We performed behavioral analyses on rooster mating patterns across two breeding cohorts with distinct sex ratios, quantifying mating frequencies and fertilization efficacy. As documented in Table 7, roosters demonstrated significant diurnal variation in mating activity (morning vs. afternoon: *p* < 0.01). The 1:8 cohort displayed higher morning mating frequency compared to the 1:10 group (*p* < 0.05), while conversely exhibiting reduced afternoon activity (*p* < 0.05).

**Figure 1:**
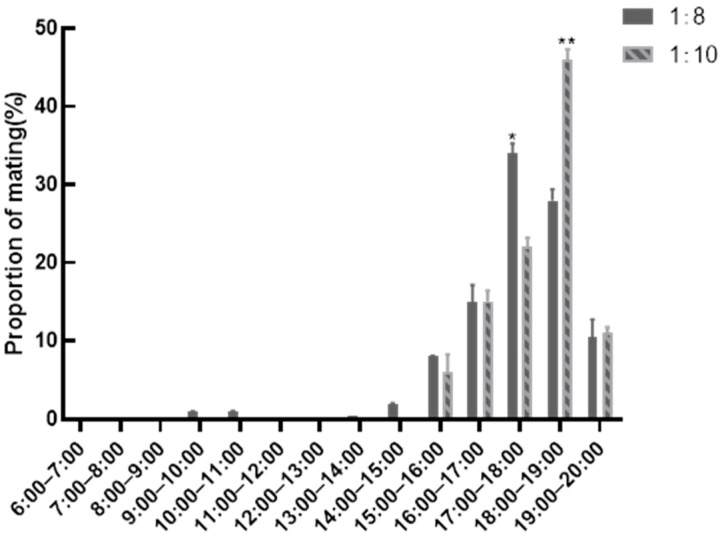
Statistics of mating frequency at different times. ** indicates very significant differences in data between the two groups (*p* < 0.01), * *p* < 0.05.

Figure 1 delineates temporal mating patterns in roosters across sex-ratio cohorts. Data analysis revealed afternoon mating predominance (16:00–19:00), peaking at 17:00–18:00. The 1:8 cohort demonstrated significantly elevated mating frequency during 17:00–18:00 versus 1:10 group (*p* < 0.01), contrasting with reduced activity at 18:00–19:00 (*p* < 0.01), indicating ratio-specific mating chronotypes.

Post-culling analysis (excluding low-performance roosters) showed enhanced reproductive efficacy in both 1:8 (*p* < 0.05) and 1:10 (*p* < 0.05) cohorts (Table 8), with adjusted ratios stabilizing at 1:8.77 and 1:10.35 without significant culling rate variations.

As detailed in Table 9, the 1:8 cohort exhibited lower mean rooster weight yet superior weight-uniformity and copulatory activity (non-significant trends, *p* > 0.05). A positive correlation emerged between weight uniformity and mating frequency across groups.

Table 10 stratifies roosters by mating performance, correlating body mass with reproductive activity. In the 1:8 cohort, 22.22% of top-performing roosters concurrently ranked in the top tertile for body mass, contrasting with twofold greater representation (55.56%) in the 1:10 cohort. The 1:10 group’s elite performers also demonstrated superior mean copulatory rates.

Comparative analysis of feeding-phase reproductive behavior in the afternoon (Table 11) revealed temporal specialization:

1:8 cohort: Peak activity concentrated at 16:00–18:00 (*p* < 0.01 vs. other intervals), with secondary elevation at 18:00–19:00 exceeding both earlier (12:00–16:00) and later (19:00–20:00) phases (*p* < 0.01);

1:10 cohort: Dominant reproductive activity spanned 16:00–19:00 (*p* < 0.01 vs. other periods), peaking at 18:00–19:00.

Intergroup comparison showed that 1:8 roosters exhibited significantly elevated 16:00–18:00 activity versus 1:10 counterparts (*p* < 0.01), with equivalent performance across the remaining intervals. 

**Table 11:** Afternoon feeding time and mating frequency statistics.

Group	12:00–16:00	16:00–18:00	18:00–19:00	19:00–20:00	*p*-Value
1:8	10.33 ± 3.79 ^C^	52.50 ± 6.25 ^Aa^	27.83 ± 13.05 ^B^	9.83 ± 7.94 ^C^	<0.001
1:10	8.17 ± 4.08 ^B^	35.67 ± 9.25 ^Ab^	41.33 ± 6.49 ^A^	15.17 ± 7.56 ^B^	<0.001
*p*-value	0.151	0.027	0.075	0.064	

^a,b^ Means in the same column with different superscripts are different (*p* < 0.05). ^A–C^ Means in the same line with different superscripts are different (*p* < 0.01).

## 4. Discussion

Male presence significantly modulates female reproductive physiology across species. In mammalian studies, androgen exposure or male introduction serves as a standard protocol for estrus induction [10]. Morphological and ethological traits of male Japanese quail (Coturnix japonica) alter female steroidogenesis and maternal resource allocation [11]. Strategic male rotation in female quail flocks enhances egg production efficiency (EPE) and fertilization capacity while modifying gonadal steroid profiles [12]. Conversely, prolonged rooster presence in layer flocks reduces hen productivity and survivorship, potentially through mating pressure attenuation and domestication-driven behavioral selection [1]. Adolescent pullets require critical pre-laying phases for ovarian maturation and energy homeostasis, where sexual maturation timing directly determines subsequent production sustainability. Notably, intermittent male exposure yields heavier females compared to continuous cohabitation [12]. Complementary studies confirm that hen presence improves rooster seminal quality [6].

Our findings demonstrate that delayed cohabitation (140-day) roosters exhibited superior body mass versus early-mixed (120-day) counterparts. The age at 50% egg production did not differ significantly between the 120 and 140 groups (156.65 vs. 155.05 days, respectively; *p* > 0.05). This phenomenon may stem from elevated mating frequency and aggressive interactions, accelerating energy expenditure and compromising metabolic reserves. The pre-laying window is vital for folliculogenesis and nutrient accrual, processes potentially disrupted by precocious mating activity and male-induced stress. However, cohabitation timing showed a negligible impact on fertilization efficiency or egg output, likely influencing primarily post-mixing weight dynamics. Crucially, fertilization success exhibited a stronger correlation with sex ratio parameters. Limited cohabitation timing effects on reproductive organ development align with prior findings that critical follicular and testicular maturation stages precede experimental timelines [13].

To address welfare concerns in intensive poultry systems, regulatory bodies and producers have established maximum stocking density (SD) standards. However, SD optimization requires breed-specific calibration due to interspecies variations and production system heterogeneity. SD critically influences growth trajectories and production metrics in modern poultry operations. Broiler studies confirm inverse correlations between SD elevation and growth performance [14,15]. Similarly, elevated SD in layers reduces egg output, potentially through SD-mediated suppression of gonadal steroids and ovarian dysfunction [16,17,18].

In natural mating colony cages, supraoptimal SD thresholds create spatial constraints that marginalize subordinate birds through restricted feeding access and nesting opportunities, ultimately impairing flock productivity. Conversely, suboptimal SD increases locomotory energy expenditure, potentially compromising production efficiency through metabolic trade-offs [19].

Our findings demonstrate that SD reduction paradoxically decreased egg output and egg weight while improving rooster weight homogeneity. This phenomenon may stem from prior uniform cage conditioning. Low-SD birds exhibited an increased amount of exercise post-transfer (more activity space), elevating hen metabolic expenditure and reducing reproductive investment. High-SD environments promoted feed particle segregation, where dominant birds preferentially consumed coarse particulates, leaving fines for subordinates—a mechanism explaining poor weight uniformity. Reduced social competition in low-SD groups likely enhanced rooster weight consistency. Notably, SD variations showed negligible impact on hatchability, potentially attributable to maintained sex ratio stability across density treatments.

The *Gallus gallus* (Red Junglefowl) naturally forms harems with dominant males maintaining 4–12 females [20]. Foundational studies caution against elevated mating ratios in conservation programs due to amplified stress biomarkers [5]. Nigerian indigenous fowl research demonstrated mating ratio independence on fertilization/hatching metrics, though nest-searching duration showed sensitivity [21]. Avian mate selection favors males exhibiting secondary sexual traits and dominance displays [22]. Higher male ratios intensify rooster social stratification, escalating combat frequency for mating priority [20], paralleling observations in geese where dominant ganders exhibit superior reproductive efficiency [23].

Our experimental data revealed that 1:9 sex ratio groups achieved higher egg production versus 1:8 cohorts (*p* < 0.05), albeit with elevated breakage rates. Breeding egg quality and mortality remained ratio-independent. The 1:8 group showed depressed fertilization rates relative to 1:10 (*p* < 0.05), potentially reflecting harem monopolization by dominant roosters and limiting subordinate mating efficacy [22]. Post-50-week analyses revealed divergent trajectories: 1:8 maintained stable fertilization versus 1:10’s decline (*p* < 0.01), and continuous mating necessitated a higher sperm volume. In future studies, interventions such as nutritional enhancement may be explored to examine their potential effects on the reproductive performance of roosters maintained under high male-to-female ratios.

Indeed, mating behavior plays a critical role in the reproductive success of animals, including poultry species [24]. As with mammals, mating behavior in poultry is not only vital for reproduction but also essential for achieving successful fertilization [25]. The reproductive success of poultry is often influenced by the sexual interactions and mating behaviors within competitive social structures [26,27,28]. Previous studies have indicated that sexual behavior is a primary factor affecting the reproductive performance of poultry [29], modulated by endocrine rhythms, oviposition timing, and microenvironment [27]. Understanding these interactions is crucial for optimizing breeding and reproductive outcomes in poultry production.

The observed concentration of mating activities during 16:00–18:00 across experimental groups corresponds with avian circadian rhythms in reproductive physiology. This temporal pattern likely optimizes gamete interaction, as morning oviposition schedules ensure ova availability for afternoon sperm–egg fusion. Furthermore, scheduled feeding protocols during this window may synchronize mating behaviors through nutrient-mediated behavioral activation. These findings emphasize the necessity of aligning husbandry practices with species-specific biological rhythms to enhance reproductive efficiency. The preference of roosters for mates and the establishment of group order after cohabitation highlight the social dynamics within poultry flocks [30]. Higher-ranking roosters, having access to more hens, exhibit increased mating activity compared to lower-ranking individuals. The observation that eliminating lower-ranking roosters significantly increased the fertilization rate suggests that the mating behavior of these individuals (lower-ranking roosters) may be unsuccessful in the presence of dominant roosters. This underscores the imperative of social structure management in breeder flocks, where selective removal of low-performance males could mitigate reproductive suppression while maintaining genetic diversity.

Breeder body mass significantly modulates fertilization efficiency. Parental-line studies demonstrate superior reproductive physiology in heavier males, characterized by enhanced testicular function, elevated androgen levels, and reduced stress biomarkers—key indicators of reproductive fitness [31]. Our findings extend this paradigm, revealing a significant positive correlation between post-50-week weight uniformity and mean daily copulatory frequency. This suggests that optimal weight uniformity facilitates consistent mating performance through improved social equilibrium, ultimately enhancing flock reproductive output. Additionally, the correlation between body weight and mating frequency sheds light on the dynamics of mating competition within the flock. Mechanistically, standard-weight individuals may avoid the dual constraints of hen resistance (toward overweight mates) and dominance challenges (from heavier competitors). Conversely, overweight roosters may face resistance from hens during mating attempts, while lighter roosters may struggle to assert their mating rights in the presence of larger, more dominant counterparts. Overall, achieving a balance in body weight distribution among roosters can play a crucial role in promoting harmonious social interactions and enhancing reproductive success in poultry flocks.

## 5. Conclusions

The 120-day mixed group outperformed the 140-day mixed group when the Lohmann Pink-shell breeder hens were kept in natural mating cages. The SEP, the body weight uniformity, and the weight control rates were all better at 694 cm^2^/bird of feeding density. The flock’s laying rate and fertilization rate were highest at a male to female ratio of 1:10, while the flock’s death and culling rates were lowest. The fertilization rate could be greatly increased by eliminating the roosters that showed no signs of mating behavior. The roosters’ enhanced copulatory conduct followed feeding, with the majority of their copulatory behavior taking place between 16:00 and 18:00. The findings of this study offer theoretical support for guiding the natural mating cage breeding of Lohmann pink-shell parental generation laying hens in China.

## Figures and Tables

**Table 1:** Age of first egg and body weight of roosters.

Group	Age of First Egg (d)	Body Weight (g)	AWG ^1^ (g)	RGR ^2^ (%)	Uniformity (%)	Control Rates (%)
120 d	156.65 ± 0.59	1909.18 ± 63.83 ^B^	93.55 ± 13.61 ^A^	5.16 ± 1.00 ^A^	96.30 ± 5.74 ^B^	107.38 ± 0.04 ^A^
140 d	155.05 ± 1.10	1972.40 ± 40.76 ^A^	28.12 ± 11.59 ^B^	1.45 ± 1.00 ^B^	98.15 ± 4.54 ^A^	101.25 ± 0.02 ^B^
*p*-value	0.055	<0.001	<0.001	<0.001	<0.001	<0.001

^A,B^ Means in the same column with different superscripts are different (*p* < 0.01). ^1^ AWG: Average weekly gain; ^2^ RGR: Relative growth rate.

**Table 2:** Effects of cohabitation age on laying performance of breeder hens (25–45 wk).

	Group	25 wk	29 wk	33 wk	37 wk	41 wk	45 wk
Laying rate (%)	120 d	96.07 ± 2.75	95.00 ± 1.23	94.90 ± 1.17	92.01 ± 3.11	90.76 ± 4.79	91.74 ± 1.87
	140 d	94.09 ± 0.91	94.30 ± 1.79	94.50 ± 1.23	91.39 ± 2.62	88.68 ± 6.44	91.04 ± 4.29
	*p*-value	0.424	0.321	0.402	0.828	0.640	0.539
Breakage rate (%)	120 d	2.25 ± 0.68	1.00 ± 0.41	0.90 ± 0.22	1.30 ± 0.81	1.54 ± 0.99	2.25 ± 1.22
	140 d	2.35 ± 0.44	1.63 ± 0.60	1.55 ± 0.40	1.98 ± 0.91	2.82 ± 1.05	2.83 ± 0.88
	*p*-value	0.574	0.061	0.052	0.240	0.055	0.457
SEP ^1^ (%)	120 d	83.32 ± 5.18	90.06 ± 4.23	93.16 ± 2.95	93.14 ± 2.47	91.37 ± 4.13	89.61 ± 3.26
	140 d	81.94 ± 3.51	89.08 ± 2.88	91.14 ± 3.35	91.90 ± 3.08	89.59 ± 6.09	89.65 ± 3.33
	*p*-value	0.621	0.456	0.436	0.425	0.324	0.756
Egg weight (g)	120 d	55.31 ± 0.34	57.50 ± 1.14	59.59 ± 0.38	59.86 ± 0.32 ^a^	60.32 ± 1.79	59.86 ± 1.37
	140 d	55.47 ± 1.04	57.43 ± 0.94	59.25 ± 0.23	58.97 ± 0.62 ^b^	59.73 ± 0.62	59.39 ± 0.95
	*p*-value	0.709	0.630	0.088	0.026	0.106	0.093
Death and culling rate of hens (%)	120 d	0	0	0	0	0	0
	140 d	0	0	0	0	0.42 ± 0.65	0
	*p*-value	-	-	-	-	0.101	-
Death and culling rate of roosters (%)	120 d	0	0	0	0	1.67 ± 4.68	0
	140 d	0	0	0	0	0	0
	*p*-value	-	-	-	-	0.070	-

^a,b^ Means in the same column with different superscripts are different (*p* < 0.05). ^1^ SEP: Percentage of settable eggs.

**Table 3:** Effect of different cohabitation ages on hatching performance (25–45 wk).

	Group	25 wk	29 wk	33 wk	37 wk	41 wk	45 wk
Fertility (%)	120 d	86.22 ± 1.44	90.56 ± 1.30	93.62 ± 2.52	92.64 ± 2.70	94.49 ± 2.32	94.71 ± 2.44
	140 d	87.58 ± 0.83	90.53 ± 0.91	93.16 ± 2.64	94.31 ± 1.77	94.44 ± 2.79	94.58 ± 1.91
	*p*-value	0.057	0.736	0.621	0.214	0.770	0.690
Hatchability (%)	120 d	80.32 ± 1.24	86.77 ± 0.64	86.25 ± 3.04 ^a^	86.03 ± 6.49	88.70 ± 5.15	87.97 ± 4.06
	140 d	81.12 ± 0.94	86.99 ± 0.31	81.47 ± 2.27 ^b^	89.42 ± 4.40	89.82 ± 3.69	87.57 ± 2.44
	*p*-value	0.081	0.772	0.025	0.370	0.594	0.841
HS ^1^ (%)	120 d	93.19 ± 0.98	95.98 ± 0.79	92.11 ± 1.12 ^A^	94.97 ± 2.99	95.60 ± 3.57	91.61 ± 4.32
	140 d	92.62 ± 0.54	96.12 ± 0.71	87.45 ± 1.83 ^B^	94.80 ± 3.95	93.54 ± 1.76	92.76 ± 3.30
	*p*-value	0.124	0.561	<0.001	0.740	0.078	0.215
HFCP ^2^ (%)	120 d	39.24 ± 1.14	42.41 ± 0.14	41.18 ± 1.15	43.25 ± 4.18	47.24 ± 3.27	44.97 ± 5.72
	140 d	36.88 ± 1.17	42.04 ± 0.11	40.34 ± 1.43	42.46 ± 6.32	43.25 ± 4.28	42.86 ± 2.13
	*p*-value	0.067	0.055	0.094	0.115	0.066	0.214

^a,b^ Means in the same column with different superscripts are different (*p* < 0.05). ^A,B^ Means in the same column with different superscripts are different (*p* < 0.01). ^1^ HS: Hatchability of settable eggs. ^2^ HFCP: healthy female chick production.

**Table 4:** Effect of different feeding densities on production performance of birds (25–45 wk).

	Group III (748 cm^2^/Birds)	Groups IV (694 cm^2^/Birds)	Group V (654 cm^2^/Birds)	Group VI (582 cm^2^/Birds)	*p*-Value
Laying rate (%)	87.84 ± 2.02 ^b^	89.42 ± 1.82 ^ab^	91.10 ± 4.94 ^ab^	92.09 ± 3.00 ^a^	0.012
Breakage rate (%)	2.28 ± 0.27	2.17 ± 0.68	2.26 ± 0.47	2.14 ± 0.44	0.341
SEP ^1^ (%)	89.99 ± 3.67 ^b^	91.89 ± 4.65 ^a^	91.03 ± 3.81 ^ab^	91.73 ± 4.35 ^ab^	0.031
Egg weight (g)	58.06 ± 0.44 ^b^	57.81 ± 0.45 ^b^	58.81 ± 0.45 ^b^	59.36 ± 0.22 ^a^	0.018
DCH ^2^ (%)	0.06 ± 0.10	0.06 ± 0.10	0.01 ± 0.02	0	0.087
DCR ^3^ (%)	0.02 ± 0.06	0	0	0.76 ± 1.31	0.064
Weight uniformity of hens	90.53 ± 4.99 ^a^	88.52 ± 4.85 ^ab^	86.89 ± 5.96 ^ab^	84.15 ± 1.67 ^b^	0.024
WCH ^4^ (%)	105.87 ± 1.57 ^b^	104.47 ± 1.60 ^b^	104.96 ± 0.92 ^b^	108.60 ± 1.95 ^a^	0.016
Weight uniformity of roosters	98.15 ± 4.54 ^a^	94.45 ± 6.09 ^ab^	94.45 ± 4.86 ^ab^	91.67 ± 4.43 ^b^	0.013
WCR ^5^ (%)	91.27 ± 2.78	89.71 ± 3.13	90.77 ± 1.14	91.71 ± 0.90	0.146
Fertility (%)	91.39 ± 1.27	91.90 ± 1.35	91.61 ± 1.39	91.72 ± 1.39	0.159
Hatchability (%)	85.10 ± 1.32	85.22 ± 0.81	85.08 ± 2.22	85.14 ± 1.93	0.715
HS ^6^ (%)	93.05 ± 0.91	92.11 ± 0.99	93.11 ± 1.51	93.37 ± 0.99	0.677
FCVR ^7^ (%)	41.83 ± 0.76	41.79 ± 0.93	41.60 ± 0.73	42.01 ± 0.91	0.482

^a,b^ Means in the same line with different superscripts are different (*p* < 0.05); ^1^ SEP: Percentage of settable eggs; ^2^ DCH: Death and culling rate of hens; ^3^ DCR: Death and culling rate of roosters; ^4^ WCH: Weight control rates of hens; ^5^ WCR: Weight control rates of roosters. ^6^ HS: Hatchability of settable eggs; ^7^ FCVR: female chick viability rate.

**Table 7:** Mating frequency differences between groups (50–51 wk).

Group	AM	PM	*p*-Value
1:8	1.24 ± 0.94 ^Ba^	98.76 ± 0.24 ^Ab^	<0.001
1:10	0.36 ± 0.32 ^Bb^	99.64 ± 0.32 ^Aa^	<0.001
*p*-value	0.017	0.035	

^a,b^ Means in the same column with different superscripts are different (*p* < 0.05). ^A,B^ Means in the same line with different superscripts are different (*p* < 0.01).

**Table 8:** Fertilization rate of eggs before and after culling.

Group	Before		After		Culling Rate (%)	*p*-Value
	Fertility (%)	Sex Ratio	Fertility (%)	Sex Ratio		
1:8	91.80 ± 0.46 ^b^	1:8.77	97.22 ± 0.69 ^a^	1:9.11	3.03 ± 0.05	0.014
1:10	90.21 ± 2.00 ^b^	1:10.35	94.84 ± 3.00 ^a^	1:10.44	3.70 ± 0.06	0.037

^a,b^ Means in the same line with different superscripts are different (*p* < 0.05).

**Table 9:** Differences in body weight and mating frequency of cocks.

Group	Average Weight (kg)	Uniformity (%)	Average Number of Mating (Times/Day)
1:8	2136.67 ± 63.14	87.27 ± 6.39	32.29 ± 2.09
1:10	2166.17 ± 78.36	85.19 ± 9.80	30.48 ± 0.99
*p*-value	0.353	0.721	0.063

**Table 10:** Weight ranking and mating frequency statistics of the top three roosters in the class within the flock.

Group	Rank Within Group			Average Number of Mating (Times/Day)		
	Repeat 1	Repeat 2	Repeat 3	Repeat 1	Repeat 2	Repeat 3
1:8	2	8	5	4.57	5.57	8.71
	6	10	6	4.00	5.43	5.57
	7	7	1	3.43	4.71	3.71
1:10	2	1	8	5.86	7.57	9.00
	9	9	2	5.43	6.43	6.57
	7	2	3	3.86	5.00	4.86

## Data Availability

Data are contained within this article.
